# Can motor competence be influenced by the type of training interventions preschool children are exposed to? A randomized experimental study comparing sports games and psychomotricity activities

**DOI:** 10.3389/fpsyg.2024.1476297

**Published:** 2024-12-23

**Authors:** Xiaodan Guo, Chuangtao Li, Zhaoxiang Zhang, Ana Filipa Silva, Filipe Manuel Clemente

**Affiliations:** ^1^Gdansk University of Physical Education and Sport, Gdańsk, Poland; ^2^Fujian Normal University, Fuzhou, China; ^3^Escola Superior Desporto e Lazer, Instituto Politécnico de Viana do Castelo, Rua Escola Industrial e Comercial de Nun’Álvares, Viana do Castelo, Portugal; ^4^Sport Physical Activity and Health Research and Innovation Center, Viana do Castelo, Portugal

**Keywords:** motor competence, motor development, child, physical exercise, motor skills

## Abstract

**Introduction:**

This study aimed to compare the effects of structured sports games (SG) and psychomotricity activities (PCM) on the locomotor, stability, and manipulative motor competencies of preschool children.

**Methods:**

A randomized controlled trial was conducted over an 8-week period, involving two experimental groups (SG, *n* = 30 and PCM, *n* = 30) and one control group (CG, *n* = 28), with participants attending two intervention sessions per week. A total of 88 5-year-old children participated in the experiment (boys *n* = 48; girls *n* = 40). They were evaluated three times (at baseline, after 4 weeks, and after 8 weeks) using the Motor Competence Assessment test to measure their locomotor, stability, and manipulative motor competencies.

**Results:**

The scores were standardized to percentiles based on sex and age. However, significant differences were observed between groups post-intervention in the locomotor domain (*p* = 0.003; $\eta_{p}^{2}$ = 0.128), with the SG showing significantly higher values compared to the CG (mean difference: 17.0%; *p* = 0.021; *d* = 0.783), and PCM (mean difference: 19.8%; *p* = 0.005; *d* = 0.947). Additionally, significant differences were found between groups post-intervention in the manipulative domain (*p* = 0.001; $\eta_{p}^{2}$ = 0.142), with the SG showing significantly higher values compared to the CG (mean difference: 19.3%; *p* = 0.009; *d* = 0.845) and PCM (mean difference: 21.4%; *p* = 0.003; *d* = 0.998).

**Discussion:**

Our study highlights the significant benefits of increased practice in developing motor competence, particularly in children’s locomotor and manipulative skills. Additionally, at this age, fun, competition, and social interaction seem to play a crucial role, as the SG group demonstrated greater improvements compared to the PCM group.

## Introduction

Motor competence (MC) refers to an individual’s ability to perform a wide range of motor skills, including both fine and gross motor activities (Stodden et al., 2008; Feitoza et al., 2022). Although it has been given different expressions to MC, such as fundamental movement skills, motor development, motor efficiency, motor coordination, motor ability or motor fitness (Cattuzzo et al., 2016), there is a broad consensus that this concept imply proficiency on fundamental movements skills (Stodden et al., 2008), which are based on locomotor, stability and manipulative abilities (Gallahue and Ozmun, 2006). Thus, MC is the overall ability to perform various motor skills, while motor efficiency is the ability to perform tasks with minimal effort, motor coordination is the smooth execution of movements, motor ability reflects innate movement potential, and motor fitness involves the physical attributes that support skilled movement (Cattuzzo et al., 2016).

The MC is fundamental to a child’s overall development, particularly during the preschool years, a critical period (between 2–3 and 6–7 years of age) for acquiring and refining these skills (Gallahue and Ozmun, 2006; True et al., 2017). MC can be linked to neural maturation and the development of motor pathways in the brain, which can be essential for coordinate movements (Gerván et al., 2017; Thomason et al., 2018). Thus, neural maturation and the development of motor pathways in the brain, particularly in areas such as the motor cortex, cerebellum, and basal ganglia, may play a critical role in MC (Doyon et al., 2009). Through motor experiences, the refinement of synaptic connections, the myelination of motor neurons, and the strengthening of corticospinal and sensorimotor pathways may contribute to enable improved coordination, precision, and motor learning (Ruddy and Carson, 2013).

High levels of MC in early childhood are associated with better physical health (Sigmundsson and Haga, 2016), cognitive function, and social–emotional well-being (Robinson et al., 2015). MC may influence cognitive skills such as memory and executive control by engaging neural networks involved in motor planning, coordination, and cognitive regulation (Fernandes et al., 2016), with evidence showing that activities requiring fine and gross motor skills enhance connectivity between the cerebellum and prefrontal cortex, thereby improving working memory, inhibitory control, and cognitive flexibility (Biino et al., 2023). This is because engaging in diverse physical activities may promote muscle strength, coordination, and motor planning (Myer et al., 2015). Furthermore, children with advanced motor skills are more likely to participate in physical activities (Wrotniak et al., 2006; Williams et al., 2008), fostering healthy lifestyle habits and enhancing their self-confidence and social interactions. This is in line with the concept of physical literacy, which highlight the need of developing motivation, confidence, physical competence knowledge, and understanding to value and engage in physical activity for life (Whitehead, 2001). Physical literacy complements motor competence by providing a broader framework that includes not only the ability to perform basic motor skills, but also the knowledge, motivation, and confidence necessary to engage in physical activities throughout life, fostering a holistic development of movement capabilities (Whitehead, 2001).

The rise in sedentary behavior among preschool children poses significant risks to their MC and overall development (Sedlak et al., 2015). A recent study found that 7-year-old children with high sedentary time spent an average of 83.8 ± 55.0 min (27.4% of their sedentary time) on screen-based activities (Hoffmann et al., 2019). Those with medium sedentary time spent 82.8 ± 50.5 min (39.8% of their sedentary time), while children with low sedentary time spent 77.2 ± 59.4 min (71.3% of their sedentary time) on screens (Hoffmann et al., 2019). Studies suggest that excessive sedentary behavior delays the development of fundamental motor skills (Zacks et al., 2021). These delays can lead to poorer MC, affecting a child’s ability to engage in and enjoy physical activities (Cantell et al., 2008). Additionally, sedentary behavior can reduce muscle strength and endurance, limiting opportunities for children to develop and refine their motor skills through active play (Kolehmainen et al., 2015). In fact, it has traditionally been assumed that the development of fundamental movement skills is guaranteed and occurs naturally. However, the literature has shown that this is not the case, with the environment (especially the practice) in which the child develops playing a significant role (Goodway and Branta, 2003; Goodway and Branta, 2003; Saraiva et al., 2024). For instance, children from lower socioeconomic backgrounds or with limited access to stimulating play environments often face reduced opportunities to engage in physical activities (Ferguson et al., 2013). The lack of physical activity not only hinders motor development but also might contribute to potential long-term health issues (Vlahov et al., 2014; Musálek et al., 2021). MC acquired in childhood may lay the foundation for lifelong physical activity patterns by fostering confidence and proficiency in movement, which promotes sustained engagement in physical activity into adulthood, ultimately contributing to better health outcomes by reducing risks of obesity, cardiovascular disease, and other chronic conditions (Petrusevski et al., 2022). Therefore, reducing sedentary behavior and promoting active play are essential for fostering optimal motor development and overall health in preschool children (Bai et al., 2024).

Introducing structured practices, even when guided and supported by feedback-driven instruction, can positively impact motor competence, especially in situations where opportunities for spontaneous practice are limited (Sutapa et al., 2021). Engaging in structured physical exercise programs can significantly impact the development of MC across locomotor, stability, and manipulative domains in preschool children (Sutapa et al., 2021). For instance, a meta-analysis has showed that physical education can significantly enhance overall MC in children and adolescents (Lorås, 2020). Some studies (van et al., 2017) suggest that regular participation in structured physical activities can enhance motor skills within these domains. The literature is unclear on how to effectively improve MC, however, exposure to (quality) practice may be a key factor, with a certain degree of variability in practice being essential to expand the child’s motor repertoire (Saraiva et al., 2024). Variability refers to the dynamic process of diversifying movement experiences to prevent repetition of fixed actions, allowing participants to engage in different ways and patterns of the same type of movement (Pesce et al., 2019). This variability may enhance learning by promoting adaptability and supporting the development of motor competence through exposure to a range of movement scenarios (Sternad, 2018). Structured programs can vary widely in content and approach. For instance, sports games and general psychomotor individual activities can influence the development of MC domains. On one hand, sports games promote team interaction, and the inherent competition serves as a stimulus to challenge and develop skills. On the other hand, general psychomotor individual activities are more individualized and do not require constant interaction with a team, allowing the individual to focus solely on the task at hand. Sports games may enhance locomotor skills through dynamic movements like running, jumping, and agility actions required during gameplay, which are engaging due to the collaborative dynamics inherent in team sports (Konoh, 2013). Manipulative skills such as throwing, catching, and kicking can be particularly promoted through strategic interactions with objects in sports games settings (Hashemi et al., 2015). In contrast, general psychomotor individual activities focus on generalized body movements, emphasizing foundational aspects of MC, can be particularly interesting for targeting specific skills such as balance exercises, fine motor tasks, and basic locomotor movements, without the competitive or cooperative demands of sports games (Teixeira Costa et al., 2015; Ene et al., 2016).

Despite the potential benefits, research on how enrollment in different physical exercise programs impacts children’s MC is limited (e.g., Stodden et al., 2008; Barnett et al., 2016). Particularly lacking are experimental studies testing how structured physical exercise programs with varying content affect MC variables in children. Conducting such experimental studies could significantly advance our understanding and guide educators in directing interventions to ensure adequate development in children’s key MC areas. Thus, this study aimed to compare the effects of structured sports games (SG) and psychomotor activities (PCM) on the locomotor, stability, and manipulative motor competencies of preschool children. It was hypothesized that locomotor and manipulative abilities will be greater in SG, while stability levels will be higher in PCM. This hypothesis is based on the expectation that the specificity of the practice plays a key role. Since SG often require multidirectional movements and coordination between hands, feet and the ball, they may have a greater impact on developing competences related to these movements. In contrast, PMC typically focuses on single-body movements, which may be more relevant for tasks that require stability, potentially offering a better transfer to stability-related tests.

## Methods

### Trial design

The objective and hypothesis of our study are directly aligned with the study design, as they provide a framework for the comparison of the effects of SG and PCM on preschool children’s motor competencies. Our hypothesis suggests that locomotor abilities will be enhanced by SG, while PCM will have a greater impact on stability. To test this hypothesis, the study utilized a randomized controlled design, assigning preschool children to three groups: SG, PCM, and a control group (CG). By measuring MC before and after the intervention using MC tests, the study design allows for a clear comparison of the effects of SG and PCM on locomotor, stability, and manipulative motor skills, thus providing evidence to support or refute the hypothesized differences in outcomes between the groups.

The study followed the CONSORT guidelines to ensure a comprehensive reporting of information (Merkow et al., 2021). The research began after approval from Yibin Third People’s Hospital under code YBSDSRMYY-2024-01. Children and their legal guardians were briefed on the study’s design, associated risks, and potential benefits. Upon their voluntary agreement to participate, legal guardians signed informed consent forms. The study adhered to ethical standards outlined in the Declaration of Helsinki.

### Participants

**Figure 1:**
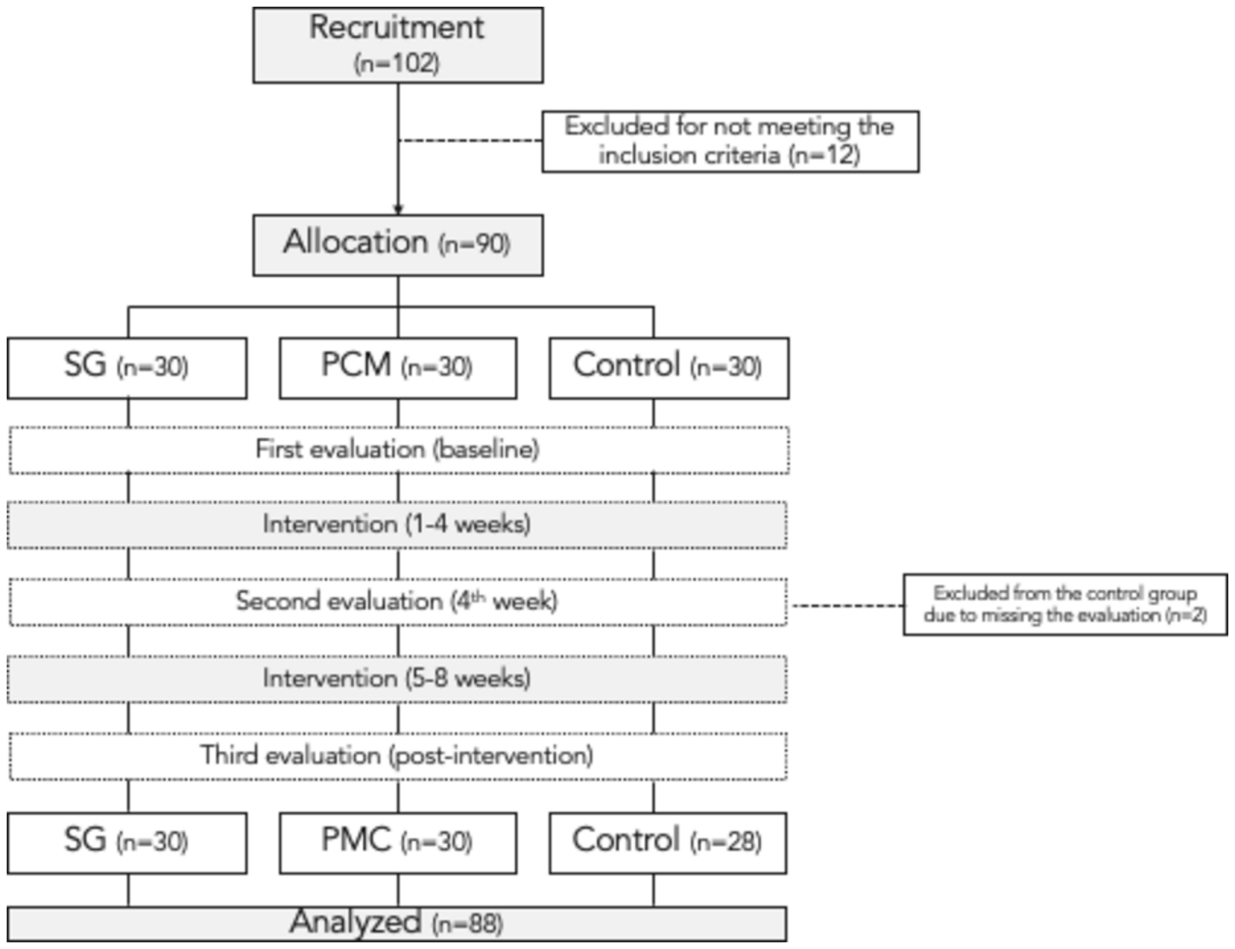
Reporting of the participants number over the phases of the experiment. SG, sports games groups; PCM, psychomotricity activities group.

The study included participants who met the following criteria: (i) healthy 5-year-old children, with no sex restrictions; (ii) not currently participating in structured sports or physical exercise programs at the time of the initial evaluation; (iii) not enrolled in other intervention programs during the study period; (iv) attending all scheduled evaluations; and (v) maintaining an adherence level of 90% or higher during the intervention. The recruitment process involved directly contacting kindergartens in the region, opting for a convenience sampling strategy because it provided easy access to the population. Through kindergarten directors, parents were invited to participate in the study, and those who expressed interest in volunteering were included in the list of potential participants (see Figure 1).

**Figure 2:**
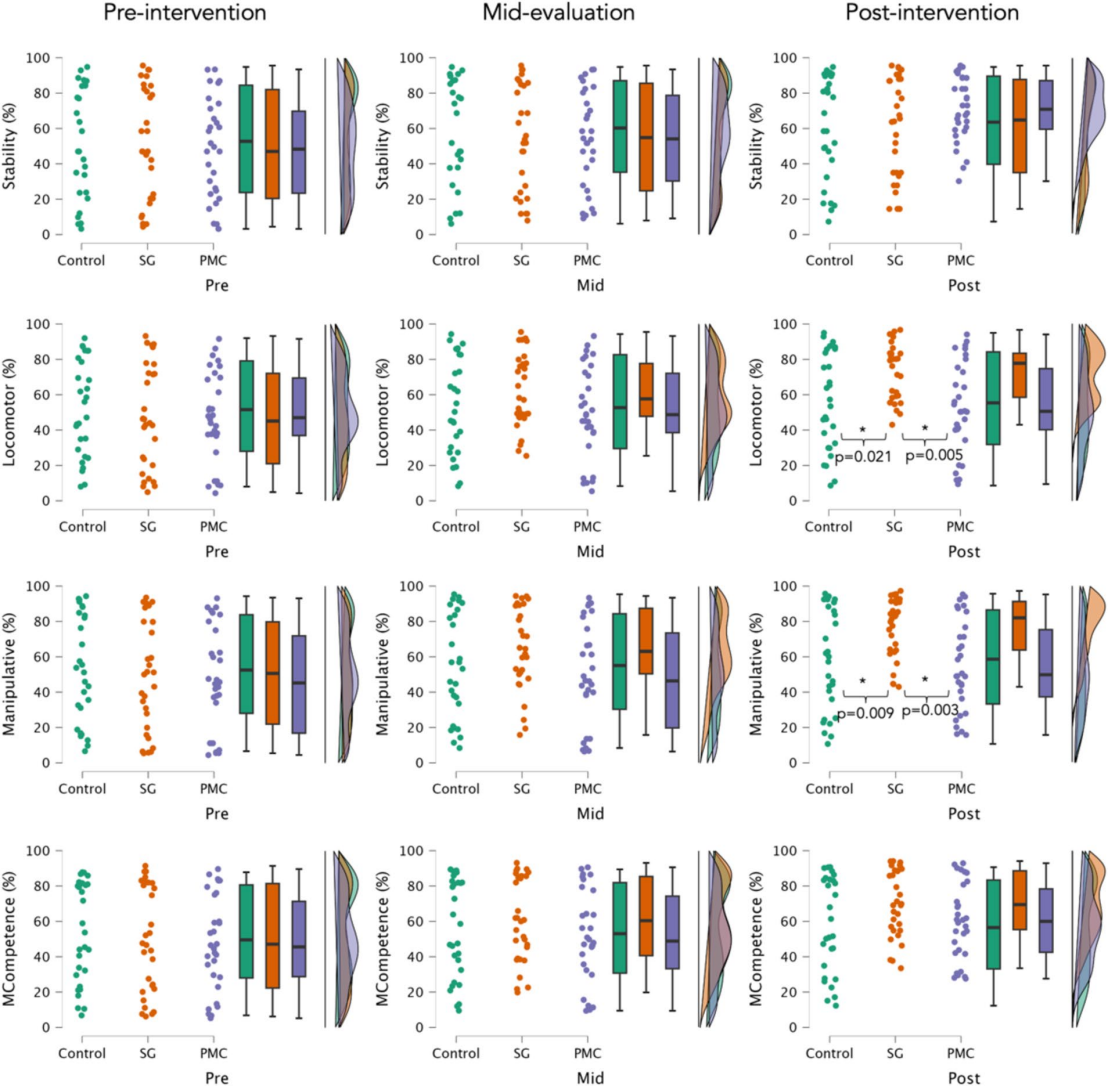
Percentiles (%) of motor competence across various domains over three evaluation periods. MC: overall motor competence score; *: statistically significant (*p* < 0.05). SG, sports games groups; PCM, psychomotricity activities group.

From an initial pool of 102 potential volunteers, 88 were considered eligible (Figure 2). The remaining 14 were excluded: 9 because they were enrolled in structured physical exercise programs at the time of the first evaluation, 3 were unavailable for measurements during the initial, and the remaining 2 were not available in the second evaluation period. The sample was composed by 48 boys 308 (110.2 ± 3.6 cm, 18.8 ± 1.7 kg) and 40 girls (108.8 ± 2.9 cm, 17.1 ± 1.7 kg).

The study lasted 10 weeks. Eight weeks were dedicated to the intervention, while the initial week was for baseline assessments, and the final week (the 10^th^) was for post-intervention evaluations. The second evaluation (intermediate) occurred in the middle of the 4th week of the intervention.

### Interventions

The interventions were conducted over 8 consecutive weeks, consisting of 30-min sessions twice a week, spaced 48 h apart. The participants had not been previously exposed to these interventions or any structured psychomotor programs. During the intervention period, the children did not participate in any additional physical education or structured physical programs. The sessions took place in kindergarten facilities and were led by the research team, who planned and prescribed the interventions. Each class (SG and PCM) was taught by two groups, each consisting of one lead teacher and two assistants, with each group specializing in only one of the approaches. The lead teachers had over 10 years of experience in children’s physical education, while the assistants had a minimum of 2 years of experience in the field. Supervision was provided in all sessions by the designated teacher and a team of monitors, who were trained in the methodological approaches for the classes and closely assisted the children in performing the activities. The contents and games of the interventions are presented in Table 1.

**Table 1:** Description of the contents for both experimental groups.

	SG	PCM
Week 1 Sessions 1 and 2	Balloon Football (10 min): Drop a balloon in the middle of a long rectangular table, and have each team try to blow the balloon towards the other team’s goal. Do not Let the Ball Drop (10 min): A teacher will stay outside the circle, observing the players. He will throw an air-filled ball, like a birthday balloon, into the middle of the circle and set a timer for 1 min or more. The goal is for the players to keep holding hands and, once the ball is in the air, not to let it fall to the ground or go outside the circle. They can use any part of their body to keep it in the air. Cooperation for the Goal! (10 min): Using a plastic ball, children grouped in teams of four will take turns running with the ball in a shuttle run to try to score in a large basket placed 50 cm from a line. The teams compete to be the fastest to score and to make the most attempts within periods of 2 min.	Leap Frog Relay (10 min): Children were divided into teams. A starting line and a finish line were marked. One child from each team hopped like a frog (using two-footed jumps) from the starting line to the finish line and back, then tagged the next teammate. The team that finished first won. Animal Movement Simon Says (10 min): “Simon Says” was played with animal movements. Commands like “Simon said crawl like a bear,” “Simon said hop like a kangaroo,” or “Simon said slither like a snake” were called out. Children only performed the action if “Simon said” first. The last child standing won. Animal Movement Freeze Dance (10 min): Music was played and children danced freely. When the music stopped, an animal movement was called out (e.g., “freeze and flap your wings like a bird!”). Children froze in that position until the music started again. This was repeated with different animal movements.
Week 2 Sessions 3 and 4	Kick the Toy! (10 min): The game starts with several benches positioned on the floor with toys placed nearby. The children stand 2 meters, and then 3 meters, away from the benches and try to hit the toys by kicking a plastic ball. Each time a toy falls down, the child scores a point. Toys in the Basket! (10 min): Many toys are positioned in the center of the court as “balls” for the children. They need to run quickly to grab a toy, then run back to the edge of the court where different baskets are positioned randomly at various distances from the starting point. Once a child scores by placing a toy in a basket, that basket is closed for him, and he must find alternative baskets to score. Run and Catch the Toy! (10 min): Grouped in teams of two, each team must pass the toy to their teammate to move forward on a small court. The team faces a single opponent who tries to intercept the toy. The goal of the team with the toy is to move forward and put the toy into a basket positioned 50 cm away from the scoring zone.	Obstacle Course Adventure (10 min): A mini obstacle course was created using climbing mats, cushions, and tunnels. Children crawled through tunnels, climbed over mats, and balanced on cushions. They were timed as they navigated the course to add a challenge and make it a fun competition. Climbing Challenge (10 min): Climbing mats were arranged against a sturdy wall to create a safe climbing wall experience. Children were encouraged to climb up and down, practicing different grips and techniques. Colored tape was used to mark paths or challenges to make it more interactive. Mat Maze Exploration (10 min): Climbing mats were laid out in a maze-like pattern on the floor. Children crawled, slithered, and weaved through the maze. They were encouraged to find different paths and explore various movements to enhance spatial awareness and agility.
Week 3 Sessions 5 and 6	Clean Up Your Room! (10 min): Many small plastic balls are scattered on the floor. Half of the participants belong to one team and are positioned on one half of the court, while the other half belong to the opposing team on the opposite side. When the teacher signals, the goal is for each team to ensure no balls remain on their side of the court. Any ball that lands on their side must be immediately thrown to the other side. The team with the fewest balls on their side when the game ends is the winner. Passing Fast and Scoring! (10 min): The game consists of groups of 4 children, positioned in a line. The ball must be successfully passed from the first to the fourth member of the group, and the fourth member must then hold the ball and throw it against a square on a wall positioned 1 meter away. The first group to complete the sequence and score against the wall earns a point. The children rotate positions in the line after each sequence is completed. Look Up the Wall! (10 min): In pairs, one child holds a tennis ball and throws it as quickly as possible against a wall. The second child must quickly catch the ball before it bounces back to the thrower.	Colorful Path Challenge (10 min): A series of colored hand and foot patterns were created on the ground using chalk or tape. Children were encouraged to follow the path by touching each colored pattern with the corresponding hand or foot. They crawled, stepped, or hopped along the path to enhance coordination and stability. Pattern Race Relay (10 min): Children were divided into teams. A starting point and a finishing point were designated with various colored patterns in between. Each team member raced to touch each pattern with alternating hands and feet (e.g., left hand on blue, right foot on red). The team that completed the course first won. Pattern Twister (10 min): The classic Twister game was adapted by replacing the colored dots with hand and foot patterns. Combinations like “left hand on yellow circle, right foot on green triangle” were called out. Children twisted and stretched to reach each pattern, testing their flexibility, coordination, and stability.
Week 4 Sessions 7 and 8	It’s Raining Balls! (10 min): Participants are grouped into teams of 4, with each team having a small basket. One member of the group will run with the basket and try to catch a plastic ball thrown into the air by the teachers. If the ball is caught directly in the basket from the air, the team earns one point. Team members will rotate the member with the basket after each attempt. Catch Your Colleague! (10 min): Half of the children have a soft small ball, while the other half do not. When the game starts, the goal is to throw the ball at children without a ball. However, if a child catches a thrown ball, they switch roles and become the one throwing the ball to catch others. Use Your Shield! (10 min): Each child will have a square of solid cardboard that they hold in their hand like a shield. Teachers will provide different soft small balls on the court, and the aim is for children to catch the balls and throw them at their colleagues. The colleagues must defend themselves using their cardboard shields.	Color Sorting Challenge (10 min): The box with colored balls was placed in the center. Children used chopsticks to pick up balls of a specific color called out by the leader or chosen randomly. They carefully maneuvered the chopsticks to transfer the balls into the corresponding sections of a divided tray or another container nearby. Race Against Time (10 min): A timer was set and children were challenged to pick up as many balls as possible within a set time limit using chopsticks. Different point values were assigned to balls of different colors for added motivation. The child with the most points at the end won the game. Obstacle Course Relay (10 min): An obstacle course was created with colored balls strategically placed along the path. Children took turns using chopsticks to pick up and transfer the balls into the box at the end of the course. Each child was timed, and they were challenged to improve their performance in subsequent rounds.
Week 5 Sessions 9 and 10	Smashing the Wall! (10 min): Different squares drawn on various levels of the walls, each with different dimensions, are set up for the game. Each child with a small soccer ball must kick as powerfully and precisely as possible to score points. Larger squares earn one point, middle-sized squares earn two points, and smaller squares earn three points. Throw It Fast! (10 min): Each child will have a basket with 5 tennis balls. At the signal, they will throw the tennis balls as quickly as possible against squares drawn on the wall. Each time a ball hits a square, one point is earned. Pass to a Friend! (10 min): Children will be positioned on the court with two other teammates to pass the ball by foot. However, teachers will try to intercept the ball, so teammates must move quickly to receive the ball and the one with the ball must pass it swiftly. Each completed pass scores one point.	Color Match Challenge (10 min): The box with colored balls was placed in the center. Children used chopsticks to pick up balls of a specific color called out by the leader or chosen randomly. They transferred each ball into a container that matched its color. Each child was timed to add a competitive edge. Chopstick Relay Race (10 min): Children were divided into teams. A series of colored balls were set up at one end of the room with empty containers corresponding to each color at the other end. One child from each team used chopsticks to pick up a ball, carry it to the matching container, and drop it in before running back to tag the next teammate. The team that finished first won. Obstacle Course Challenge (10 min): An obstacle course was created with colored balls placed along the path. Children navigated the course while using chopsticks to pick up the balls and place them into a central box or container at the end. Each child was timed individually or challenged to complete the course without dropping any balls.
Week 6 Sessions 11 and 12	Pass to Your Captain! (10 min): Two groups of 3 children each will face off against each other. In each group, one child will be designated as the captain, positioned at the endline of a small court. The objective of each team is to advance the ball forward using only hand passes (no dribbling allowed), aiming to successfully pass to their captain to score one point. Choose Fast! (20 min total, split into 10 min segments): Children on the field will move across the court either with the ball at their feet or in their hands, dribbling (moving the ball forward by dribbling, receiving, and dribbling again). When a color is named at the signal, they must quickly maneuver the ball to reach the area of the court designated with that color. The activity is split into two 10-min segments: one for ball control with the feet and another for ball control with the hands.	Footprint Jumping Race (10 min): A large circle was drawn on the ground with single or double footprints placed inside and outside the circle. Children took turns standing outside the circle and jumped from one footprint to the next, following a designated path around the circle. Each child was timed to determine who completed the circle the fastest. Follow the Footprints (10 min): Single and double footprints were scattered randomly inside and outside the circle. Instructions were called out for children to follow (e.g., “Jump to the double footprint inside the circle!”). Children listened carefully and jumped to the correct footprint as instructed. The challenge was increased by adding more complex sequences as they progressed. Footprint Memory Game (10 min): Pairs of single or double footprints were placed randomly inside and outside the circle. Children took turns flipping over two cards (representing footprints) to find matching pairs. When a match was found, they jumped to those footprints inside or outside the circle. The player with the most matches at the end of the game won.
Week 7 Sessions 13 and 14	Suicides with Foot Control! (10 min): Children with ball control at their feet will compete to complete the suicide drill as quickly as possible. After the signal, they will go 3 meters, return to the start line, go 5 meters, return again, and then go 7 meters and return once more. They must maintain control of the ball at all times. Pass to Your Captain! (10 min): In this game, two groups of 3 children each will compete against each other. Each group designates one child as the captain, positioned at the endline of a small court. The objective is for each team to advance the ball forward using only passes with their feet (no dribbling allowed). Successfully passing the ball to their captain scores one point. Suicides with Hand Control! (10 min): Children with ball control in their hands will compete in the suicide drill. Upon the signal, they will attempt to complete the drill by going 3 meters, returning to the start line, proceeding 5 meters, returning again, and then covering 7 meters before returning once more. They must maintain control of the ball throughout the drill.	Tape Maze Challenge (10 min): Colored tape was used to create a maze pattern on the floor indoors or on a paved area outdoors. Dead ends and multiple paths leading to the exit were ensured. Children navigated through the maze, aiming to find their way to the exit as quickly as possible. Each child was timed to add a competitive element. Obstacle Course Maze (10 min): An obstacle course was set up indoors with various objects such as chairs, tables, cushions, and cardboard boxes. Paths between these obstacles formed a maze-like structure. Children navigated through the course, going around or under obstacles to reach the exit. The difficulty was adjusted based on the children’s age and skill level. Natural Outdoor Maze (10 min): Natural elements such as bushes, trees, and rocks in a garden or park were used to create a maze. A clear entrance and exit point were marked. Children explored the maze, making decisions at intersections and dead ends to find the correct path to the exit. This activity encouraged outdoor exploration and helped develop problem-solving skills.
Week 8 Sessions 15 and 16	Smashing the Wall! (5 min): Different squares drawn on various levels of the walls, each with different dimensions, are set up for the game. Each child with a small soccer ball must kick as powerfully and precisely as possible to score points. Larger squares earn one point, middle-sized squares earn two points, and smaller squares earn three points. Throw It Fast! (5 min): Each child will have a basket with 5 tennis balls. At the signal, they will throw the tennis balls as quickly as possible against squares drawn on the wall. Each time a ball hits a square, one point is earned. Pass to Your Captain! (10 min): In this game, two groups of 3 children each will compete against each other. Each group designates one child as the captain, positioned at the endline of a small court. The objective is for each team to advance the ball forward using only passes with their feet (no dribbling allowed). Successfully passing the ball to their captain scores one point. Pass to Your Captain! (10 min): In this game, two groups of 3 children each will compete against each other. Each group designates one child as the captain, positioned at the endline of a small court. The objective is for each team to advance the ball forward using only passes with their feet or hands (no dribbling allowed). Successfully passing the ball to their captain scores one point.	Climbing Wall Challenge (10 min): Climbing mats were installed against a sturdy wall to create a safe climbing wall experience. Colorful holds or stickers were placed at different heights to encourage children to climb up and down using various grips and techniques. This activity promoted upper body strength, coordination, and spatial awareness as they navigated the climbing wall. Balance Beam Adventure (10 min): Climbing mats were laid out in a row to create a balance beam path on the floor. Children practiced walking, hopping, and balancing along the beam, adjusting their speed and movements to maintain balance. This helped improve core strength, stability, and proprioception (awareness of body position). Obstacle Course Exploration (10 min): Climbing mats, cushions, tunnels, and other soft obstacles were arranged to create an indoor obstacle course. It included crawling under tunnels, climbing over mats, and balancing on cushions. Children navigated the course, developing full-body strength, coordination, and spatial awareness as they maneuvered through different challenges.

SG: sports games groups; PCM: psychomotricity activities group.

During the initial week of assessments, the research team met with the participants and conducted an unregistered pilot class to ensure the children became familiar with the teachers. The pilot session involved introductions and some ice-breaker recreational activities, aiming to establish a social foundation for the upcoming intervention phase. The session was the same for both experimental groups, ensuring similar conditions.

### Outcomes

In each of the evaluation weeks, morning sessions were dedicated to conducting data assessments consistently across three evaluations. The evaluations were conducted in one-hour blocks, starting at 9 a.m., followed by 10 a.m. and 11 a.m. Participants were grouped together to facilitate efficient assessment in a circuit format. The evaluations at each time point were standardized for all participants, ensuring consistency in the timing and conditions, as well as the same day of the week for each session. This approach helped to ensure the replicability of the evaluations. These assessments were conducted indoors in a controlled environment, specifically at a temperature of 21.5°C and a relative humidity of 55%. A team of six evaluators administered the Motor Competence Assessment tests. Each evaluator received 2 weeks of training on the specific tests they were responsible for, and a pre- and post-test for accuracy was conducted in a pilot phase to ensure the quality of the observations. Children were assessed in groups, following a structured sequence that included demographic information, anthropometric measurements, and a standardized warm-up routine. This warm-up involved 5 min of jogging followed by 3 min each of upper and lower limb dynamic stretching. The assessment battery then included tests for jumping sideways, shifting platforms, ball throwing velocity, ball kicking velocity, standing long jump, and shuttle run change of direction. This approach ensured that all evaluations were conducted under consistent conditions, providing a reliable basis for data collection and analysis. The motor competence assessment battery was chosen because it has been validated (Luz et al., 2016) and showed reliable for evaluating MC in children (Silva et al., 2022). The tests were followed by 2-min rest periods, during which the children had the chance to relax and interact with their peers. Teachers offered verbal encouragement to maintain their motivation. Additionally, motivation was further enhanced by presenting the tests as challenges, designed to encourage their commitment to the evaluations.

### Shifting platforms

Participants in this test started by standing on one of two wooden platforms, each measuring 25 cm by 25 cm by 2 cm, and supported by four 3.7 cm feet at the corners (Rodrigues et al., 2019). The second platform was positioned adjacent to them on the floor, either to their right or left, depending on convenience. Upon hearing the command “Ready and Go,” participants swiftly moved the adjacent platform to the opposite side and stepped onto it. This sequence was repeated as quickly as possible for 20 s. Each successful transition earned participants two points—one for moving the platform and another for stepping onto it. The test demonstrated excellent reliability with an intra-class correlation score of 0.99 (Silva et al., 2022). Each participant was allowed two attempts, with a 3-min rest period between them, and only the highest score from the two trials was recorded. The average within-participant coefficient of variability for the completed trials was 1.4%.

### Jumping sideways

Participants were tasked with performing sideways jumps using both feet simultaneously for a duration of 15 s on a rectangular surface measuring 100 cm long and 60 cm wide, divided by a small wooden beam (60 cm long, 4 cm high, and 2 cm wide) (Rodrigues et al., 2019). The test began with the prompt “Ready and Go.” Points were awarded for each successful jump where both feet landed within the designated area without touching the boundaries or stepping on the wooden beam. Each participant completed two trials, with only the highest score recorded. The assessment demonstrated strong reliability, achieving an intra-class correlation coefficient of 0.84 (Silva et al., 2022). A preliminary familiarization trial was provided for practice purposes but did not count towards the final score. To ensure participants were adequately rested between attempts, a 3-min rest period separated each trial.

### Ball throwing velocity

Participants stood behind a designated line positioned 1 meter above the floor, facing a wall located at least 6 meters away in a designated area measuring at least 5 meters by 5 meters (Rodrigues et al., 2019). The objective was to throw a ball against the wall using an overarm motion at maximum speed. Before each throw, participants were allowed one or two preparatory steps. A cross measuring 40 cm by 40 cm was placed on the wall at a height of 170 cm to assist with alignment. The type of ball used was a tennis ball (6.5 cm diameter, 57 g weight). Peak ball velocity was measured in meters per second using a shoulder-level radar gun (Pocket radar, Model PR1000-BC, Inc. Santa Rosa, California) positioned near the throwing line facing the wall. This radar gun had previously demonstrated concurrent validity against the Stalker Radar (reference criterion), establishing its reliability and sensitivity for measuring ball velocity during both throwing and kicking activities (Hernández-Belmonte and Sánchez-Pay, 2021). The test achieved a high level of reliability, indicated by an intra-class correlation score of 0.86 (Silva et al., 2022). Participants were permitted two attempts, and the highest score was recorded. A preliminary trial was provided for familiarization purposes but did not contribute to the final score. Between each trial, participants were given a 1-min rest period to minimize fatigue and ensure consistent performance. The average within-participant coefficient of variability for the completed trials was 3.2%.

### Ball kicking velocity

Participants positioned themselves beneath a 1-meter line in an area with dimensions of at least 5 meters by 5 meters, ensuring a minimum distance of 6 meters from a wall (Rodrigues et al., 2019). The task involved kicking a soccer ball at maximum velocity against the wall. Before each kick, participants were allowed one or two preparatory balancing steps. The size of the soccer ball was 62 cm circumference and 350 g weight.

Ball velocity was measured in meters per second using a radar gun (Pocket radar, Model PR1000-BC, Inc. Santa Rosa, California) positioned beside the participant’s dominant foot, near the 1-meter line on the floor, and facing the target wall. The test demonstrated very good reliability with an intra-class correlation score of 0.86 (Silva et al., 2022). Participants were allowed two attempts, with only the best score recorded. A preliminary familiarization trial was provided but not included in the final scoring. Between trials, participants had a 1-min rest period to minimize fatigue and ensure consistent performance. The average within-participant coefficient of variability for the completed trials was 4.7%.

### Standing long jump

Participants began the test by positioning themselves on a designated starting line and were instructed to perform a maximal bilateral jump, executing a simultaneous take-off and landing with both feet (Rodrigues et al., 2019). The jump spanned over a marked surface, or a measuring tape placed perpendicular to the starting line alongside the jumping area. To aid in accuracy, a flexible scale tape was placed on the floor to mark the landing spot where the back of the heel closest to the starting line touched down. Children were instructed to propel themselves forward, ensuring that any imbalance that occurred would be in the forward direction while keeping their feet planted on the ground. If they lost balance backward, the jump was repeated. The distance in centimeters from the starting line to this point was then recorded as the measurement of the jump. The test demonstrated excellent reliability with an intra-class correlation score of 0.97 (Silva et al., 2022). Participants were given two attempts, and the best result was considered for scoring purposes. A 1-min rest interval was allocated between each trial to ensure optimal performance and minimize any potential fatigue effects. The average within-participant coefficient of variability for the completed trials was 3.8%.

### Shuttle run change-of-direction test

Participants began positioned on a 100 cm long and 5 cm wide start line (Rodrigues et al., 2019). Upon hearing the command “Ready and Go,” they sprinted at maximum speed towards another 100 cm long and 5 cm wide line located 10 meters away. The starting position was standardized, with participants consistently using their preferred leg for both starting and changing direction. Initially, participants started in a staggered stance with their preferred leg forward, ensuring this positioning was recorded to maintain consistency across all evaluations. Immediately past this line, two rounded blocks (each 10 cm high and 5 cm in diameter) were placed, spaced 25 cm apart. Participants retrieved one block, sprinted back to the starting line, and placed it beyond the line, disregarding its exact position. They then returned to collect the second block. The test concluded once participants crossed the start/finish line while carrying the second block. Timing was measured using two pairs of photocells positioned at hip height (Wichro, Wireless Race, Chronojump Boscosystem, Spain). Each participant completed two trials, with a 2-min rest interval between them. The test exhibited very good reliability, boasting an intra-class correlation score of 0.86 (Silva et al., 2022). The final score was determined by the best time achieved from the two trials. The average within-participant coefficient of variability for the completed trials was 4.1%.

### Secondary outcomes

To mitigate potential influences of physical activity and screen time on the outcomes, we employed a custom survey adapted from the Surveillance of Digital-Media Habits in Early Childhood Questionnaire, focusing on screen media use (Susilowati et al., 2021). Additionally, parents completed a validated physical activity questionnaire specifically designed for preschool children, comprising six items. These surveys were administered to parents during the initial, intermediate, and final evaluations to ensure comprehensive monitoring throughout the study period (Gascón et al., 2013).

### Sample size

The initial exploration of the recommended sample size involved examining an effect size (f) of 0.3, aiming for a power of 0.95 with 3 groups and 3 measurements. Using G*Power software (version 3.1.9.6, Universität Düsseldorf, Psychologie – HHU, Düsseldorf, Germany), an estimation for a repeated measures ANOVA with within-between interaction suggested a recommended total sample size of 39 participants.

### Randomization

Randomization was achieved by assigning numbers to participants and then randomly allocating them to one of the groups using opaque envelopes, ensuring an equal chance of enrollment in each group. A 1:1 allocation ratio was ensured. The randomization process was conducted manually by a researcher who had no further interaction with the participants during subsequent stages of the study. Group allocation occurred prior to the initial assessment of MC, and no participants changed groups throughout the study. Neither the evaluators nor the children were blinded to the study.

### Statistical procedures

Transforming participants’ results in each MCA test into age- and sex-specific normative values (percentiles) was crucial for calculating MCA sub-scales and total scores, as detailed in previous studies (Rodrigues et al., 2021). These studies identified significant developmental changes in the relationships among model components, consistently representing three domains: locomotor, stability, and manipulative sub-scales. Furthermore, recent research (Rodrigues et al., 2022) emphasized the averaging of normative values across age and sex categories for each test to compute MCA sub-scales and total scores. The formula applied for calculating each sub-scale (e.g., locomotor, stability, manipulative) was ((LC test 1 / (LC test 1 + LC test 2)) * P test 1) + ((LC test 2 / (LC test 1 + LC test 2)) * P test 2), where LC denotes loading coefficient and P denotes percentile value. This approach ensured robust evaluation and interpretation of MC across diverse demographic groups.

Descriptive statistics, including mean and standard deviation, were computed initially. The outliers were initially assessed using a Q-Q plot, and no significant deviations were observed. Normality as well as homogeneity were confirmed using Kolmogorov–Smirnov and Levene’s tests, respectively, indicating normality (*p* > 0.05) and homogeneity (*p* > 0.05) within the sample. A mixed ANOVA was subsequently conducted, incorporating time (baseline, 3-months, and 6-months) and groups (target games, striking/fielding sports, net/wall games, invasion games, and a CG) to explore potential significant interactions. Effect size was assessed using partial eta squared ($\eta_{p}^{2}$), and post-hoc analysis was performed using the Bonferroni test. The standardized effect size for pairwise comparisons was calculated using Cohen’s d, with the magnitude of differences interpreted as follows (Hopkins et al., 2009): 0.0–0.2 indicating trivial effects, 0.2–0.6 indicating small effects, 0.6–1.2 indicating moderate effects, and 1.2–2.0 indicating large effects. Statistical analyses were carried out using SPSS (IBM Corp. Released 2021. IBM SPSS Statistics for Windows, Version 28.0. Armonk, NY: IBM Corp.), with statistical significance set at *p* < 0.05.

## Results

Table 2 presents the anthropometric characteristics (body mass and height) of boys and girls from each sub-group.

**Table 2:** Descriptive statistics (mean ± standard deviation) of the body mass and height of boys and girls from each sub-group.

	CG (*n* = 28)		SG group (*n* = 30)		PCM group (*n* = 30)	
	Boys (*n* = 14)	Girls (*n* = 14)	Boys (*n* = 19)	Girls (*n* = 11)	Boys (*n* = 15)	Girls (*n* = 15)
Body mass (kg)	19.0 ± 1.3	17.6 ± 1.7	19.0 ± 1.7	16.7 ± 1.7	18.5 ± 2.1	16.9 ± 1.7
Height (cm)	109.7 ± 2.8	108.7 ± 2.8	110.5 ± 4.2	108.3 ± 3.1	110.3 ± 3.6	109.2 ± 2.9

Figure 2 shows percentiles (%) of MC across various domains over three evaluation periods. Significant interactions between time and groups were observed in the stability (*F* = 23.797; *p* < 0.001; $\eta_{p}^{2}$=0.359), locomotor (*F* = 28.820; *p* < 0.001; $\eta_{p}^{2}$=0.404), manipulative (*F* = 26.206; *p* < 0.001; $\eta_{p}^{2}$=0.381), and overall MC score (*F* = 20.012; *p* < 0.001; $\eta_{p}^{2}$=0.320).

No significant differences were found between groups in the baseline in stability (*F* = 0.244; *p* = 0.784; $\eta_{p}^{2}$=0.006), locomotor (*F* = 0.289; *p* = 0.750; $\eta_{p}^{2}$=0.007), manipulative (*F* = 0.319; *p* = 0.728; $\eta_{p}^{2}$=0.007), and MC overall score (*F* = 0.284; *p* = 0.754; $\eta_{p}^{2}$=0.007). Additionally, no significant differences were found between groups in the intermediate evaluation in stability (*F* = 0.095; *p* = 0.910; $\eta_{p}^{2}$=0.002), locomotor (*F* = 1.747; *p* = 0.180; $\eta_{p}^{2}$=0.039), manipulative (*F* = 2.201; *p* = 0.117; $\eta_{p}^{2}$=0.049), and MC overall score (*F* = 1.011; *p* = 0.368; $\eta_{p}^{2}$=0.023).

No significant differences were found between groups post-intervention in stability (*F* = 1.552; *p* = 0.218; $\eta_{p}^{2}$=0.035), and overall MC score (*F* = 2.474; *p* = 0.090; $\eta_{p}^{2}$=0.055). However, significant differences were observed between groups post-intervention in the locomotor domain (*F* = 6.220; *p* = 0.003; $\eta_{p}^{2}$=0.128), with the SG showing significantly higher values compared to the CG (mean difference: 17.0%; *p* = 0.021; *d* = 0.783), and PCM (mean difference: 19.8%; *p* = 0.005; *d* = 0.947). Additionally, significant differences were found between groups post-intervention in the manipulative domain (*F* = 7.059; *p* = 0.001; $\eta_{p}^{2}$=0.142), with the SG showing significantly higher values compared to the CG (mean difference: 19.3%; *p* = 0.009; *d* = 0.845) and PCM (mean difference: 21.4%; *p* = 0.003; *d* = 0.998).

The Table 2 shows the descriptive statistics (mean ± standard deviation) of the scores standardized to percentiles obtained by participants in the three groups across the three evaluation periods. Significant interactions between groups and time were observed in jumping sideways (*F* = 19.478; *p* < 0.001; $\eta_{p}^{2}$=0.314), shifting platforms (*F* = 14.497; *p* < 0.001; $\eta_{p}^{2}$=0.254), standing long jump (*F* = 30.522; *p* < 0.001; $\eta_{p}^{2}$=0.418), shuttle run (*F* = 18.520; *p* < 0.001; $\eta_{p}^{2}$=0.304), throwing velocity (*F* = 14.255; *p* < 0.001; $\eta_{p}^{2}$=0.251), and kicking velocity (*F* = 25.714; *p* < 0.001; $\eta_{p}^{2}$=0.377). No significant differences were found between groups in post-intervention performance for jumping sideways (*F* = 0.797; *p* = 0.454; $\eta_{p}^{2}$=0.018), shifting platforms (*F* = 2.595; *p* = 0.081; $\eta_{p}^{2}$=0.058), and shuttle run (*F* = 2.807; *p* = 0.066; $\eta_{p}^{2}$=0.062). However, significant differences were observed in the standing long jump (*F* = 8.504; *p <* 0.001; $\eta_{p}^{2}$=0.167), throwing velocity (*F* = 3.731; *p* = 0.028; $\eta_{p}^{2}$=0.081) and kicking velocity (*F* = 9.205; *p <* 0.001; $\eta_{p}^{2}$=0.178).

Specifically, in the standing long jump, the SG group showed significantly higher values compared to the CG (mean difference: 39.4%; *p* = 0.006; d = 0.070), although no significant differences were found when comparing SG to PCM (mean difference: 49.5%; *p* > 0.999; *d* = 0.060). In throwing velocity, the SG group exhibited significantly higher values compared to the PCM group (mean difference: 33.4%; *p* = 0.036; d = 0.718). Finally, in kicking velocity, the SG group showed significantly higher values than the CG (mean difference: 41.9%; *p* = 0.002; *d* = 1.044) (see Table 3).

**Table 3:** Descriptive statistics (mean ± standard deviation) of the motor competence percentiles obtained by participants in the three groups across the three evaluation periods.

	CG (*n* = 28)	SG (*n* = 30)	PCM (*n* = 30)	Differences between the groups
Jumping sideways (%)				
Baseline	51.4 ± 33.7^#,$^	46.1 ± 34.1^#,$^	42.4 ± 39.7^#,$^	*F* = 0.552; *p* = 0.578; $\eta_{p}^{2}$=0.013
After 4-weeks	55.4 ± 33.3^*^	51.5 ± 33.0^*,$^	48.6 ± 30.0^*,$^	*F* = 0.324; *p* = 0.724; $\eta_{p}^{2}$=0.008
Post-intervention	58.9 ± 32.1^*^	56.4 ± 31.3^*,#^	65.5 ± 21.8^*,#^	*F* = 0.797; *p* = 0.454; $\eta_{p}^{2}$=0.018
Dif. Post-Bas (%)	14.6	22.3	54.5	
Shifting platforms (%)				
Baseline	54.7 ± 31.4^#,$^	55.3 ± 31.2^#,$^	52.4 ± 29.1^#,$^	*F* = 0.074; *p* = 0.929; $\eta_{p}^{2}$=0.002
After 4-weeks	58.7 ± 30.8^*^	60.0 ± 29.6^*,$^	58.8 ± 36.7^*,$^	*F* = 0.017; *p* = 0.983; $\eta_{p}^{2}$<0.001
Post-intervention	61.9 ± 29.0^*^	64.7 ± 27.9^*,#^	75.8 ± 15.7^*,#^	*F* = 2.595; *p* = 0.081; $\eta_{p}^{2}$=0.058
Dif. Post-Bas (%)	13.2	17.0	44.7	
Standing long jump (%)				
Baseline	53.0 ± 29.3	51.1 ± 32.0^#,$^	47.3 ± 29.6	*F* = 0.265; *p* = 0.767; $\eta_{p}^{2}$=0.006
After 4-weeks	54.1 ± 29.3	64.1 ± 24.3^*,$^	49.3 ± 29.8	*F* = 2.185; *p* = 0.119; $\eta_{p}^{2}$=0.049
Post-intervention	54.8 ± 29.2^b^	76.4 ± 15.5^a,c,*,#^	51.1 ± 29.9^b^	*F* = 8.504; *p <* 0.001; $\eta_{p}^{2}$=0.167
Dif. Post-Bas (%)	3.4	49.5	8.0	
Shuttle run (%)				
Baseline	51.1 ± 27.3	43.5 ± 28.3^#,$^	47.2 ± 27.8^$^	*F* = 0.546; *p* = 0.581; $\eta_{p}^{2}$=0.013
After 4-weeks	53.8 ± 27.7^$^	59.4 ± 22.0^*,$^	50.6 ± 28.3^$^	*F* = 0.873; *p* = 0.421; $\eta_{p}^{2}$=0.020
Post-intervention	56.8 ± 27.7^#^	69.2 ± 19.6^*,#^	54.9 ± 27.8^*,#^	*F* = 2.807; *p* = 0.066; $\eta_{p}^{2}$=0.062
Dif. Post-Bas (%)	11.2	59.1	16.3	
Throwing velocity (%)				
Baseline	53.7 ± 32.6	50.6 ± 31.1^#,$^	46.4 ± 32.1^$^	*F* = 0.381; *p* = 0.684; $\eta_{p}^{2}$=0.009
After 4-weeks	55.5 ± 31.9	62.0 ± 24.9^*,$^	48.2 ± 32.5^$^	*F* = 1.599; *p* = 0.208; $\eta_{p}^{2}$=0.036
Post-intervention	57.5 ± 31.1	72.3 ± 21.7^c,*,#^	54.2 ± 28.7^b,*,#^	*F* = 3.731; *p* = 0.028; $\eta_{p}^{2}$=0.081
Dif. Post-Bas (%)	7.1	42.9	16.8	
Kicking velocity (%)				
Baseline	52.3 ± 32.5	47.9 ± 33.7^#,$^	46.9 ± 30.7^$^	*F* = 0.223; *p* = 0.801; $\eta_{p}^{2}$=0.005
After 4-weeks	53.8 ± 32.1	65.4 ± 23.3^*,$^	49.5 ± 30.5^$^	*F* = 2.446; *p* = 0.093; $\eta_{p}^{2}$=0.054
Post-intervention	57.1 ± 31.1^b^	81.0 ± 14.7^a,c,*,#^	56.4 ± 27.2^b,*,#^	*F* = 9.205; *p <* 0.001; $\eta_{p}^{2}$=0.178
Dif. Post-Bas (%)	9.2	69.1	20.3	

a: significantly different from the CG (*p* < 0.05); b: significantly different from the SG group (*p* < 0.05); c: significantly different from PCM group; *: significantly different from baseline (*p* < 0.05); #: significantly different from mid-evaluation (*p* < 0.05); $: significantly different from post-intervention (*p* < 0.05); Dif. Post-Bas (%): percentage of difference between baseline and post-intervention (within-group).

### Secondary outcomes

Table 4 presents the time spent on digital media during weekdays and the result of the parent questionnaire on physical activity levels in baseline and post-intervention in the CG, SG and PCM groups.

**Table 4:** Percentage of the time spent on digital media during weekdays and on sedentary and moderate to vigorous activities resulting from the parent questionnaire on physical activity levels in the baseline and post-intervention in each sub-group.

	CG (*n* = 28)		SG (*n* = 30)		PCM (*n* = 30)	
	Baseline	Post-intervention	Baseline	Post-intervention	Baseline	Post-intervention
Time spent on digital media during weekdays (%)						
Less than 3 h of exposure	48	51	51	49	46	48
3–5 h of exposure	33	28	32	27	35	31
5–8 h of exposure	19	21	17	24	19	21
Parent questionnaire on physical activity levels (%)						
Sedentary activities	84	82	86	79	83	77
Moderate to vigorous activities	16	18	14	21	17	23

## Discussion

The aim of the present study was to evaluate the impacts of structured SG and PCM on the locomotor, stability, and manipulative motor skills of preschool-aged children. Our study’s findings suggest that interventions based on sports games significantly enhanced the main MC domains of locomotor and manipulative skills, resulting in significantly improved scores among children. These interventions were also found to be significantly more effective compared to both the PCM group and the CG. Additionally, it was revealed that neither experimental group (SG and PCM) differed significantly from the CG in terms of stability and overall MC scores after the intervention period.

Our research has showed that children participating in SG, particularly those focusing on mastering ball control and learning team dynamics, as well as targeting through kicking and throwing at specific goals, experienced significant benefits in improving motor skills related to locomotion. These improvements significantly enhanced abilities such as standing long jump and shuttle run. The statistical significance of these improvements was observed after 8 weeks of training in comparison to PCM and CG. Interestingly, no differences were found in the 4^th^ week. Skill acquisition is a dynamic process influenced by the frequency, intensity, and specificity of practice (Smith et al., 1999). By employing various SG drills and drawing on Newell’s constraints model (Newell, 1986), the interaction among individual constraints (e.g., physical capabilities like strength and coordination), task constraints (e.g., specific movement demands of team sports), and environmental constraints (e.g., dynamic and unpredictable game scenarios) likely created opportunities for learners to enhance locomotor capacities such as explosive running power, directly contributing to improvements in tasks like the standing long jump. Additionally, the variability inherent in open drills promoted a broader range of movement patterns and responsiveness, potentially translating into better performance in the shuttle run test.

Our study substantiates specificity principles by indicating that targeted training over an 8-week period led to statistically significant improvements in motor skills, contrasting with the absence of such improvements observed in the initial 4-week phase. Possibly, the initial practice stages focus on cognitive processing and perceptual-motor mapping before transitioning to automaticity through continued training may justify the findings (Ackerman and Cianciolo, 2000). Furthermore, the observed benefits can be attributed to neuroplastic changes within the motor cortex, as repeated engagement in specific motor tasks refines neural pathways responsible for movement coordination and efficiency (Calmels, 2020). Considering the improvements observed in the SG group, they can possibly be attributed to the integration of complex movements such as running, jumping, and directional changes in dynamic, real-time scenarios. These activities emphasize specific motor skills essential for locomotion. This practice may translate into enhanced performance in locomotor assessments, whereas general psychomotor interventions might not offer the necessary task-specific focus required for improving locomotor skills (Jiménez-Díaz et al., 2019). Furthermore, the competition and cooperation promoted by SG may have provided a greater stimulus for improvement, as well as the constant manipulation of objects, such as the ball.

In our experimental study, significant improvements in manipulative MC were observed in the SG experimental group, particularly in ball kicking and throwing velocities. Post-intervention scores showed a significant enhancement compared to both the PCM and control groups. These differences became statistically significant only after 8 weeks, with no differences at the 4-week. This outcome highlights the specific advantages of SG training interventions in developing precise motor skills necessary for manipulating objects in dynamic environments, such as throwing and kicking towards targets (McNeill et al., 2020). SG require participants to engage in specific motor tasks such as kicking and throwing with precision and timing, demanding the integration of sensory feedback and motor planning in dynamic settings (Davids et al., 2000). Moreover, these games can provide children with immediate feedback, allowing them to adapt their behavior and coordination in dynamic settings. Skills learned in contexts closely resembling actual performance conditions (i.e., SG) are more likely to transfer effectively to similar tasks compared to skills acquired through general psychomotor exercises which can explain the improvements in the tests (Soderstrom and Bjork, 2015). Thus, the refinement of motor programs involved in precise manipulative tasks like ball kicking and throwing, potentially explaining the observed improvements in the SG group (Mukherjee et al., 2017). In contrast, general PCM interventions may lack the specificity and intensity needed to elicit similar enhancements in manipulative MC, focusing instead on broader aspects of sensory integration and coordination.

Our study did not find any significant effects of either experimental group (SG and PCM) in enhancing stability MC compared to the CG. SG often prioritize dynamic movements actions such as running, jumping, and rapid changes in direction, which may not directly translate to improvements in stability tasks. In contrast, general PCM interventions typically encompass activities aimed at enhancing overall body awareness and sensorimotor integration, although potentially lacking the specificity required to improve static balance skills specifically (Moschos and Pollatou, 2022). Improvements in stability MC are closely linked to adaptations in proprioceptive feedback mechanisms and neuromuscular coordination (Wong et al., 2012), which may require targeted exercises focusing explicitly on balance and postural control. Indeed, it should be noted that the regulatory mechanisms of postural balance depend on the interaction between the visual, vestibular and proprioceptive systems, which depend on the maturational process (Paillard and Noé, 2015). Nevertheless, from the age of 4, children undergo rapid development related to the maturation of the central nervous system involved in integrating sensory inputs to maintain balance (Venetsanou and Kambas, 2011), for instance, the strengthening of corticospinal connections enhances motor coordination domains such as precision and timing in voluntary movements, critical for tasks requiring dynamic balance and stability. Therefore, the observed lack of significant effects in our study could be a result of a maturated stability system, and/or could also reflect the complexity of assessing stability MC, which may necessitate longer intervention periods or more focused training protocols than those provided in the current study duration. On the other hand, future studies could consider incorporating different exercises, such as children’s yoga or more targeted balance exercises.

While our study suggests the beneficial impact of SG interventions on enhancing locomotor and manipulative MC among children, several limitations should be noted. One limitation is the lack of monitoring the specific dose of exposure to drills and exercises for each participant during practices, making it impossible to quantify the necessary dose magnitude for observing improvements. Additionally, heterogeneity within the group, such as differences in sex or competence levels, can also influence the magnitude of adaptations. Also, MC is a multifaceted concept that can vary depending on the observed abilities, potentially yielding different effects with alternative battery tests. A consensus must be established in the future to define appropriate assessment batteries and standardize key definitions, which will help strengthen and clarify the findings in the field. Therefore, future research should employ multiple assessment batteries and increase sample size and diversity to ensure the generalizability of findings. Finally, an 8-week period may not be sufficient to gain a comprehensive understanding of the long-term effects or to identify potential sensitive periods or plateaus resulting from the programs; therefore, longer intervention durations are necessary.

Despite its limitations, our study is among the few that explore the effects of different intervention approaches on preschool children aimed at enhancing their MC. Experimental studies are crucial, particularly because a significant portion of research in this field consists of observational studies, primarily cross-sectional. Furthermore, it is recommended that interventions of varying durations be implemented to better understand how prolonged exposure may influence group differences. Our results highlight a key message: activities such as SG can significantly enhance locomotor and manipulative competencies. Moreover, they can be particularly beneficial for preschoolers as they may promote the development of social skills through teamwork and communication, enhance emotional regulation by teaching children to manage frustration and celebrate collective successes, and stimulate cognitive growth by encouraging problem-solving and strategic thinking, making them a valuable complement to regular psychomotor teaching classes. However, a more comprehensive range of interventions should include PCM and others that improve stability (e.g., balance training, coordination). Therefore, interventions should always be fitted to the individual needs of children and encompass a wide array of activities to target all dimensions of MC effectively. Moreover, it is important to note that the interventions used are flexible in terms of the equipment required for implementation, making them applicable in a variety of settings. However, all interventions, including SG, must be properly supervised by teachers to ensure that competitiveness does not disrupt the learning environment. Additionally, incorporating more cooperative games can help children enjoy the experience.

## Conclusion

In conclusion, our study emphasizes the significant benefits of SG interventions in enhancing children’s MC, particularly in locomotor and manipulative domains. Over an 8-week period, participants engaging in SG activities showed significant improvements in skills such as shuttle run, throwing, and kicking, which translated into enhanced performance in specific motor assessments compared to psychomotor and control groups. These findings highlight the effectiveness of targeted, task-specific training in refining motor skills. However, enhancing stability remains a different challenge, as none of the experimental interventions showed effective in improving this competency.

## Data Availability

The raw data supporting the conclusions of this article will be made available by the authors, without undue reservation.
